# Relationships between Specific Airway Resistance and Forced Expiratory Flows in Asthmatic Children

**DOI:** 10.1371/journal.pone.0005270

**Published:** 2009-04-17

**Authors:** Bruno Mahut, Ludovic Trinquart, Plamen Bokov, Muriel Le Bourgeois, Serge Waernessyckle, Claudine Peiffer, Christophe Delclaux

**Affiliations:** 1 Cabinet La Berma, Antony, France; 2 Assistance Publique-Hôpitaux de Paris, Hôpital Européen Georges Pompidou, Département de Physiologie, Radio-Isotopes, Clinique de la Dyspnée, Paris, France; 3 Assistance Publique-Hôpitaux de Paris, Hôpital Necker Enfants-Malades, Service de Pneumo-Allergologie Pédiatrique, Paris, France; 4 Université Paris Descartes, Paris, France; 5 Assistance Publique-Hôpitaux de Paris, Hôpital Européen Georges Pompido, Unité de Recherche Clinique et d'Epidémiologie, Paris, France; 6 Laboratoire Matière et Systèmes Complexes (MSC); UMR 7057 CNRS & Université Paris Diderot, Paris, France; 7 Mosquito Respiratory Research Group, Paris, France; 8 INSERM CIE 4, Paris, France; University of Giessen Lung Center, Germany

## Abstract

**Background:**

The earliest changes associated with airflow obstruction in asthmatic children are a proportionally greater reduction in FEF_50%_ than in FEV_1_ using spirometry, and an increase in specific airway resistance (sRaw) using body plethysmography. Consequently, we hypothesized that sRaw could be better linked to FEF_50%_ than to FEV_1_. The first aim was to assess the relationships between forced expiratory flows and sRaw in a large group of asthmatic children in a transversal study. We then performed a longitudinal study in order to determine whether sRaw of preschool children could predict subsequent impairment of forced expiratory flows at school age.

**Methodology:**

Pulmonary function tests (sRaw and forced expiratory flows) of 2193 asthmatic children were selected for a transversal analysis, while 365 children were retrospectively selected for longitudinal assessment from preschool to school age.

**Principal Findings:**

The transversal data showed that sRaw is differently related to FEF_50%_ (−1/sRaw) and to FEV_1_ (near linearly). These results were further explained by a simple one-compartment lung model, which justified the shape of the observed relationships. As hypothesized, sRaw correlated more strongly to FEF_50%_ than to FEV_1_ (r = −0.64 *versus* −0.39, respectively; p<0.001). In the longitudinal part of the study, sRaw at preschool age correlated with subsequent FEF_50%_ (% predicted) (−0.31, 95% CI, −0.40 to −0.22), but weakly with subsequent FEV_1_ (% predicted) (−0.09, 95% CI, −0.20 to 0).

**Conclusion:**

Specific Raw is more strongly related to FEF_50%_ than to FEV_1_ and could be used in preschool children to predict subsequent mild airflow limitation.

## Introduction

The description of airway resistance (Raw) measurement is absent from recent international recommendations of pulmonary function tests in both adults [1] and children [2]. This lack of interest for Raw measurements might be explained by the fact that the resistance of airways is widely considered to be dependant of large airway calibre, since it is assumed that contribution of small airways, i.e. less than 2 mm, represents only 10% of the total amount of resistance [3].

However, plethysmography is widely used by paediatric pulmonologists and important therapeutic decisions are based on this approach. The measurement of airway resistance, and its relationship with lung volume, was first described by Dubois and colleagues [4], [5]. They detailed the two steps of the procedure, namely assessment of simultaneous variations of respiratory flow and variations in plethysmographic volume (ΔV′/ΔVpleth), followed by the measurement of thoracic gas volume. Subsequently, Dab and Alexander showed that the measurement of thoracic gas volume can be omitted, especially in children who do not tolerate this step [6], [7]. The measure obtained by this single step being the specific airway resistance (sRaw). Surprisingly, little is known about the strength and the nature of the relationships between specific airway resistance and forced expiratory flows, two commonly used methods in lung function assessment.

The earliest change associated with airflow obstruction is thought to be a slowing in the terminal portion of the spirogram, even when the initial part of the spirogram is barely affected. It is reflected in a proportionally greater reduction in the instantaneous flow measured at 50% of forced vital capacity (FEF_50%_) than in forced expiratory volume at 1 second (FEV_1_). Evidencing this mild impairment is problematic in young children since even trained children may not meet all international criteria for spirometry [8].

Previous prospective studies in which asthmatic patients were followed up from school-age to adulthood showed that the lung function phenotype was acquired early in childhood [9], [10]. Along this line, it has been demonstrated that specific Raw was altered in persistent wheezers as early as 5 years of age [11]. This result suggests that sRaw is a sensitive marker of lung function impairment.

We therefore hypothesized that if sRaw is a sensitive marker of airway obstruction, it would be more closely linked to FEF_50%_ than to FEV_1_. The first aim of this study was to assess the relationships between forced expiratory flows and specific Raw in a large group of asthmatic children in a transversal study. To further validate our hypothesis, we also performed a longitudinal study in order to determine whether specific Raw of preschool children could predict subsequent impairment of forced expiratory flows at school age.

## Methods

### Patients

Our Institutional Review Board of the French learned society for respiratory medicine – Société de Pneumologie de Langue Française waived the need for informed consent accordingly to French Law due to the observational character (part of routine practice) of the study (patients prospectively included in the La Berma cohort were informed of data collection, see below) [12].

#### Transversal assessment

All asthmatic patients (7 to 18 years old) referred to La Berma medical office (prospective cohort of asthmatic children) or to the Necker Enfants Malades hospital for lung function tests (LFT) between 2002 and 2007 were identified retrospectively. The diagnosis of asthma was based on symptoms of recurrent episodes of airflow obstruction or airway hyperresponsiveness, airflow obstruction has been shown to be at least partially reversible, and alternative diagnoses were excluded. We selected LFT that included both sRaw and spirometry measurements under antiasthma treatment. Only one LFT per child was selected, thus when several LFT were available, we selected that with the lowest sRaw value (and the corresponding spirometry data).

#### Longitudinal assessment

Successive LFT performed before (preschool) and after 7 years of age in the La Berma cohort were analysed. Due to the retrospective design of the study and the inherent variability of LFT in asthmatic children, all the LFT performed under treatment during these two periods were eligible and the mean value of each functional parameter was calculated.

### Lung function tests

#### Plethysmographic measurement of specific airway resistance

Specific airway resistance was measured using a whole-body plethysmograph (Sensormedics 2000 Autobox system; MSR, Pantin, France) during panting (frequency 100/min±20) that facilitates opening of the glottis [4]. Median value of sRaw, as determined by the slope of the line between the points where the flow reaches 0.5 L/s (sRaw_0.5_), of ten technically satisfactory specific resistance loops was collected, as previously described [13].

#### Spirometry

Three technically acceptable measurements were performed. The two end of test criteria were those defined by the ATS/ERS task force [14]. Reference values were based on equations edited by Zapletal [15].

### One-compartment lung model

To further validate the observed relationships between sRaw and forced expiratory flows, a simple one-compartment model was evaluated in order to assess whether the theoretical relationships between sRaw and forced expiratory flows fitted the observed relationships.

We used a one-compartment model based on the mechanical-electric equivalence:

1. an expansible part, characterized by a respiratory system compliance (C_RS_) and an initial volume (total lung capacity: TLC), is analogue to an electric condenser characterized by a capacity (C) and an initial charge (Q_i_)
2. a resistive part, characterized by an airway resistance (Raw), is analogue to an electric resistance (R).

During a forced expiratory manoeuvre, the lung is initially inflated to the volume TLC, then a volume equivalent to the vital capacity (VC) is expired till the residual volume (RV), corresponding to the final charge Q_f_.

The equation that governs the electric discharge of a capacitor through a resistance is: 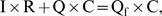 where I = dQ/dt and Q is the electric charge. The solution is:
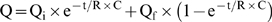
which is equivalent to the following equation with lung parameters:
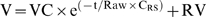
where VC is the vital capacity and RV the residual volume and so the expiratory flow, which is the speed of discharging the lung volume is:
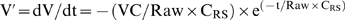
So the FEV_1_ is:
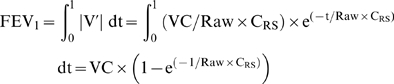
(1)and:
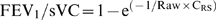
The FEF_50%_ is:
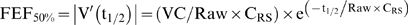
(2)where t_1/2_ is the lag time for discharge of half of the vital capacity, which can be expressed as follows: 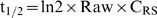
and finally, the FEF_50_ can be expressed as:
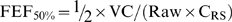
To further assess the validity of our model, observed data were fitted with the following equations (see below, statistical analyses):
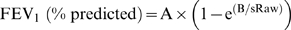
(1')and
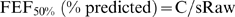
(2')where A, B and C are constants, independent on height and sex.

### Statistical methods

Results are expressed as median [interquartile range]. We assessed the correlation of sRaw_0.5_ with FEV_1_ and FEF_50%_ by computing the two corresponding Spearman rank correlation coefficients and associated 95% confidence intervals. The Spearman coefficient is a distribution-free measure of association (it is the Pearson correlation coefficient applied to the rank-ordered variables, with average ranks assigned to ties). We further tested equality between those two dependent Spearman correlation coefficients, in order to compare the strengths of association between sRaw_0.5_ and FEV_1_, and between sRaw_0.5_ and FEF_50%_.

Non-linear curves were fitted through the scatter plots relating sRaw_0.5_ with FEV_1_ and FEF_50%_, respectively based on the equations (1') and (2') introduced above in the Methods. Parameter values of the best-fit curve were estimated using non-linear least squares. We provided an initial guesstimate for the two parameter values.

We evaluated the optimal cut point values of sRaw_0.5_ which best separated patients with FEV_1_<80% from others, and patients with FEF_50%_<60% from others. We used the minimum p-value approach: all observed values of sRaw_0.5_, except the top and bottom five percent of the extremes values in the data, were examined as candidates for the cut point. The value was chosen that best separated patient outcome according to a maximum chi-squared statistic and minimum p-value based on the two by two contingency table crossing the categorized sRaw_0.5_ and expiratory flow rate. Because of multiple testing and inflation in the type I error rate, we used the Miller-Siegmund p-value adjustment formulae. Analyses were performed using SAS and S-Plus softwares. A p-value<0.05 was considered significant.

## Results

### Relationships between sRaw_0.5_ and forced expiratory flow rates

By checking all medical records of the period ranging from 2002 to 2007, we retrieved 2193 consecutive asthmatic patients who had a LFT that included both sRaw_0.5_ and spirometric measurements (Table 1).

**Table 1:** Clinical and functional characteristics of the asthmatic children.

Characteristic	
Median [interquartile]	
Number of patients	2193
Sex ratio, girls/boys	841/1352
age, years	10.8 [9.0–12.8]
height, cm	144 [135–155]
weight, kg	36 [30–47]
sRaw_0.5_, kPa.s	0.76 [0.62–0.97]
FEV_1_, % predicted	99 [88–108]
FEV_1_/FVC, %	84 [79–88]
FEF_25–75%_, % predicted	82 [66–97]
FEF_50%_, % predicted	82 [66–99]

There was a significantly higher correlation between specific Raw_0.5_ (raw values or predicted) and FEF_50%_ (% predicted) than between sRaw_0.5_ and FEV_1_ (percentage of predicted values or raw values adjusted for age and height) (Table 2).

**Table 2:** Relationship between sRaw_0.5_ and forced expiratory flow rates as assessed by Spearman's signed rank test.

Variable	Correlation with sRaw_0.5_ (95% confidence interval)	Partial correlation with sRaw_0.5_ (95% confidence interval)*
FEV_1_, L	−0.14 (−0.18 to −0.10)	−0.37 (−0.40 to −0.33)
FEV_1_, % predicted	−0.39 (−0.43 to −0.36)	−0.39 (−0.43 to −0.36)
FEF_25–75%_, L/s	−0.49 (−0.52 to −0.46)	−0.63 (−0.66 to −0.61)
FEF_50%_, L/s	−0.47 (−0.50 to −0.43)	−0.56 (−0.59 to −0.53)
FEF_50%_, % predicted	−0.64 (−0.67 to −0.62)	−0.64 (−0.67 to −0.62)
FEV_1_/FVC, %	−0.59 (−0.62 to −0.56)	−0.59 (−0.62 to −0.56)
FEF_25–75%_/FVC ratio**	−0.18 (−0.22 to −0.14)	−0.18 (−0.22 to −0.14)

* Spearman correlation coefficient, data adjusted for age and height.

** FEF_25–75%_/FVC ratio is a crude index of airway size relative to lung size [18].

Correlation coefficients are calculated for expiratory flow rates expressed in terms of both absolute and of % predicted values (left column), as well as in terms of values adjusted for age and height (right column) showing that for the latter we obtained very similar results to those obtained for values expressed in % predicted.

**Figure 1:**
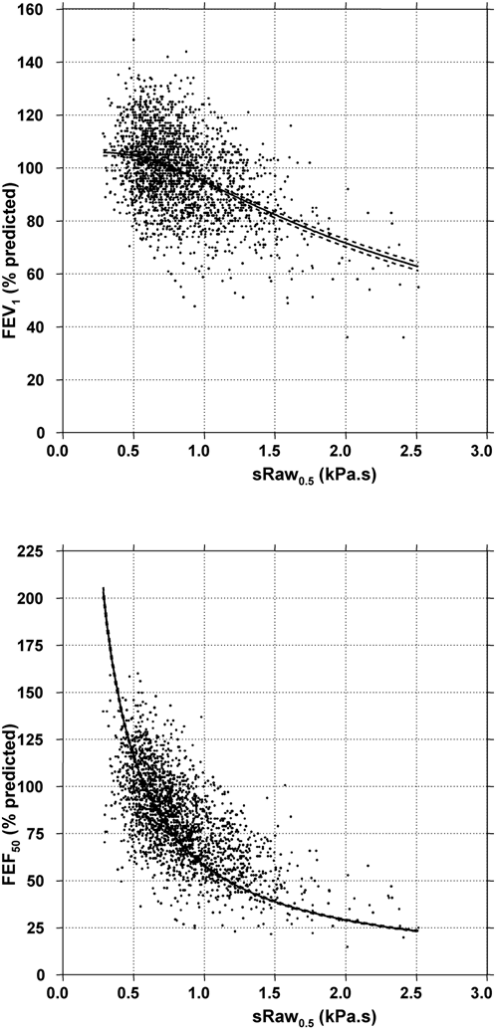
Relationships between sRaw and forced expiratory flows: observed data and non-linear regression modelling. Non-linear regression modelling showed that the curves based on our model equations (1') and (2') (see equations in the Methods) fitted well the scatter plots sRaw_0.5_ for both FEV_1_ (upper panel) and FEF_50%_ (lower panel), respectively.

**Figure 2:**
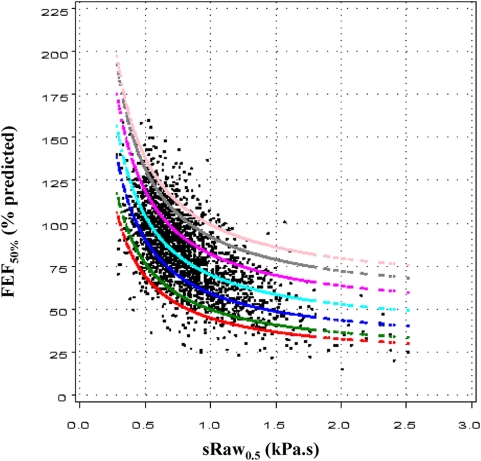
Quantile regression models in which a specified conditional quantile (or percentile) of FEF_50%_ was expressed as a linear function of the inverse of sRaw_0.5_. The 5% (red line), 10% (green line), 25% (dark blue line), 50% (blue), 75% (dark pink line), 90% (grey line) and 95% (pink line) percentiles are shown. When using quantile regression by examining multiple percentiles rather than only the mean, one can examine how the entire distribution of FEF_50%_ changes with sRaw_0.5_.

Non-linear regression modelling showed that the curves based on our model equations (1') and (2') fitted the scatter plots, despite the wide range of expiratory flows for a given sRaw_0.5_ (Figure 1). The mathematical function relating sRaw_0.5_ to FEF_50%_ was different from that relating sRaw_0.5_ to FEV_1_ (1/R and near linear, respectively). As shown in Figure 1 and 2, there was a large scatter of FEF_50%_ and FEV_1_ values for each given value of sRaw_0.5_. However, the latter was similar over the whole range of sRaw_0.5_ values and the mathematical relationships equally fitted the subgroups of centiles when analysed separately (Figure 2). These results were confirmed when analysing LFTs from both centers separately.

### Longitudinal assessment of the predictive value of sRaw_0.5_ for subsequent impairment of forced expiratory flow rates

Among patient referred to La Berma medical office, preschool LFT (less than 7 year-old patients) and subsequent LFT (more than 7 year-old) were available for 365 children (Table 3). The mean number of LFT in this cohort was 6.6 during the follow-up.

**Table 3:** Clinical and functional characteristics of the asthmatic children included in the longitudinal part of the study.

Characteristic, Median [interquartile]	Preschool age (<7 yrs)	School age (>7 yrs)
Number of LFT	2 [1]–[4]	3 [1]–[6]
Sex ratio, girls/boys	134/231	134/231
Age, years	6.0 [5.0–6.0]	9.0 [8.0–10.0]
sRaw_0.5_, kPa.s	0.72 [0.59–0.88]	0.83 [0.70–0.99]
FEV_1_, % predicted	NA	101 [91–110]
FEV_1_/FVC, %	NA	83 [79–86]
FEF_25–75_, % predicted	NA	79 [67–91]
FEF_50_, % predicted	NA	79 [67–91]

NA: spirometry was not available in preschool children.

Specific Raw_0.5_ at preschool age correlated with subsequent sRaw (Spearman correlation coefficient 0.47, 95% CI, 0.39 to 0.55), with subsequent FEF_50%_ (% predicted) (−0.31, 95% CI, −0.40 to −0.22), but weakly with subsequent FEV_1_ (% predicted) (−0.09, 95% CI, −0.20 to 0).

A preschool airway resistance superior to 1.35 kPa.s was associated with subsequent FEF_50%_<60% (relative risk 1.14, 95% CI 1.07 to 1.22, p<0.001) but not with subsequent FEV_1_<80% (relative risk 1.03, 95% CI, 0.98 to 1.08, p = 0.26).

### Theoretical relationships between forced expiratory flows and airway resistance

**Figure 3:**
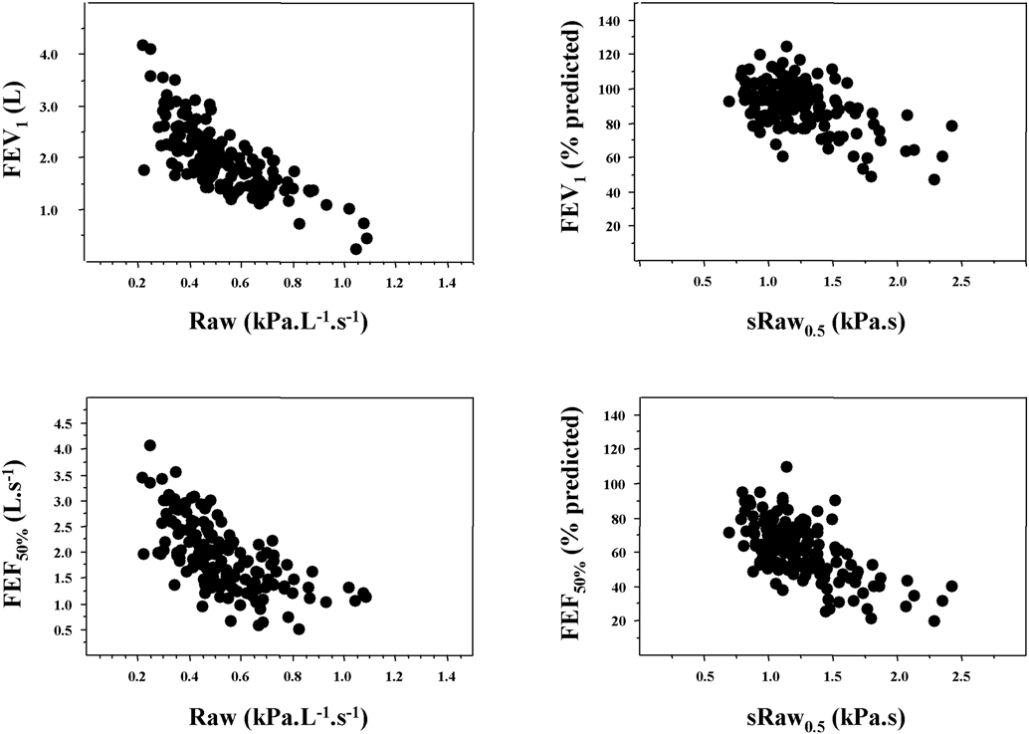
Relationships between sRaw or Raw and forced expiratory flows. Both measurements of sRaw_0.5_ and Raw_0.5_ have been obtained in a subgroup of 161 patients (La Berma Cohort). Left side: relationships between Raw (raw values) and forced expiratory flows (raw values: FEV_1_, upper panel, FEF_50%_, lower panel). Right side: relationships between sRaw (raw values) and forced expiratory flows (% predicted: FEV_1_, upper panel, FEF_50%_, lower panel). The figures show the similarity of the shape of the observed relationships that all were statistically significant (p<0.0001 for the four relationships; FEV_1_: linear regression; FEF_50%_: logarithmic regression).

In order to better explain the fact that our model predicted the relationships between raw values of expiratory flows and airway resistance while we evaluated in patients the relationships between predicted values of expiratory flows and sRaw, we show the observed relationships in a subgroup of children between raw values of Raw and forced expiratory flows (FEV_1_, FEF_50%_) in figure 3 (left side). Airway resistance is inversely proportional to thoracic gas volume [5], while sRaw, corresponding to the product of Raw by thoracic gas volume, is independent of height. Predicted values of expiratory flow rates are related to the height of children and therefore on their lung volumes [15]. Consequently, we expressed the relationships between raw values of sRaw and expiratory flows expressed as percentage of their predicted values. Figure 3 shows that whatever the modality of expression of the relationships (Raw and flows expressed as raw values, or sRaw [raw value] versus flows [% predicted]) similar shapes were observed.

## Discussion

Our main result is to show that on the opposite of a common belief, sRaw is more closely related to FEF_50%_ than to FEV_1_ and is appropriate to detect early airway obstruction in children. The validity of our results is further supported by a simple on-compartment model. Using two study designs, i.e. transversal and longitudinal, we demonstrate that sRaw was more closely linked to FEF_50%_ than to FEV_1_ that may explain why sRaw can detect mild levels of airway obstruction.

There was a large scatter of FEF_50%_ and FEV_1_ values for each given value of sRaw_0.5_, especially in the normal range, and one may wonder whether the observed relationships were related to underlying physical properties linking resistance and flow, which can be considered as cousins rather than brothers. Along this line, figure 3 shows that there was also a large variability of the relationships between Raw (raw values) and forced expiratory flows. It is necessary to emphasize that sRaw is not a true resistance but a dynamic viscosity (expressed as kPa.s), which incorporates the thoracic gas volume (∼end-expiratory lung volume) that may increase in asthmatic patients and add further variability between sRaw and flows [16]. However, the relationship between sRaw and FEF_50%_ is better in children with impaired forced expiratory flows, confirming the ability of the two approaches to similarly detect airway obstruction. The lower variability of the relationships in children with obvious airway obstruction is in accordance with the results of Guyatt and Alpers who concluded that specific airway conductance and FEV_1_ are fairly well related in subjects with airway obstruction but there was no apparent relationship in normal subjects [17]. In this latter study involving 752 men (498 current smokers), the authors suggested that log value of specific conductance is a more sensitive index of airway obstruction in early chronic bronchitis than FEV_1_ [17].

To further explain the shape of the relationships between sRaw and FEF_50%_ on one hand and sRaw and FEV_1_ on the other hand, we deliberately chose the simplest model, a R–C circuit in series. Our assumption was that the simplest lung model would be the most appropriate model, provided it can describe the observed functional relationships. Obviously this model does not take into account many physiological confounders such as changes in recoil (and hence the compliance value), airway wall properties (and hence the resistance) and choke point during forced expiration. The fact that the observed relationships were fitted by equations obtained from the model further suggest that, while not equivalent, both sRaw (raw value) and forced expiratory flows (expressed as % of predicted values) may evaluate similar levels and sites of airway obstruction.

In conclusion, our study shows that preschool sRaw measurements are more closely related to FEF_50%_ than to FEV_1_ suggesting that sRaw measurements in early childhood may contribute to predict mid-expiratory flow limitation at school-age, which is of clinical relevance in children not able to perform spirometry. Our results may argue in the favour of the come back of plethysmographic measurement in international recommendations.
